# Inflammatory Myofibroblastic Tumor of the Breast

**DOI:** 10.1155/2015/705127

**Published:** 2015-02-12

**Authors:** Christos Markopoulos, Petros Charalampoudis, Evangelia Karagiannis, Zoh Antonopoulou, Dimitrios Mantas

**Affiliations:** The Breast Unit, 2nd Propedeutic Department of Surgery, Medical School, National University of Athens, 17 Agiou Thoma Street, 11527 Athens, Greece

## Abstract

Inflammatory myofibroblastic tumors (IMTs) of the breast represent extremely rare lesions. Due to the scarcity of reports, their natural history, recurrence, and metastatic potential remain poorly defined. We report on a case of a primary breast IMT in a postmenopausal female patient treated successfully with breast conserving surgery and review the literature pertaining to this rare entity.

## 1. Introduction

Inflammatory myofibroblastic tumors have largely been considered as a subgroup of inflammatory pseudotumors [1] and have been encountered in various anatomical locations [2–7]. Interestingly, mammary IMTs have scarcely been reported. These lesions demonstrate variable clinical features and their neoplastic nature is ill-defined to date. Recent studies on IMTs have identified clonal abnormalities of the anaplastic lymphoma kinase (ALK) gene, but the impact of this feature on the neoplastic behavior of the tumor is not clarified [8]. We herein report on a rare case of a primary breast IMT in a postmenopausal woman and review the literature regarding the clinicopathological characteristics of these extremely rare lesions.

## 2. Case Presentation

**Figure 1:**
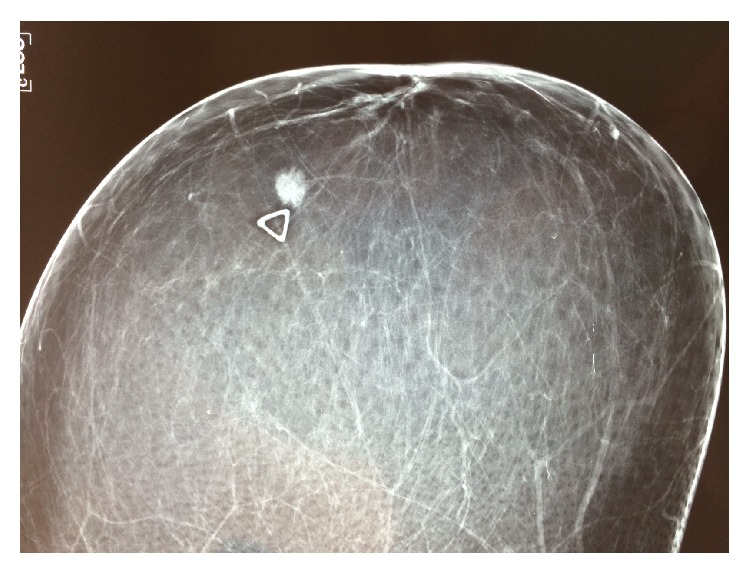
Left craniocaudal (CC) mammographic view: round centimetric mass with indistinct margin (arrow); BI-RADS 4.

**Figure 2:**
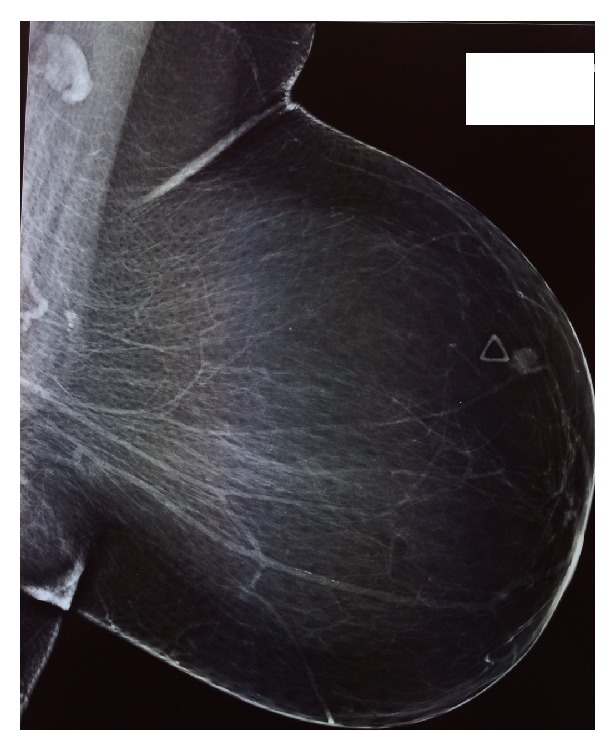
Left mediolateral oblique mammographic view: the mass appears superficial.

**Figure 3:**
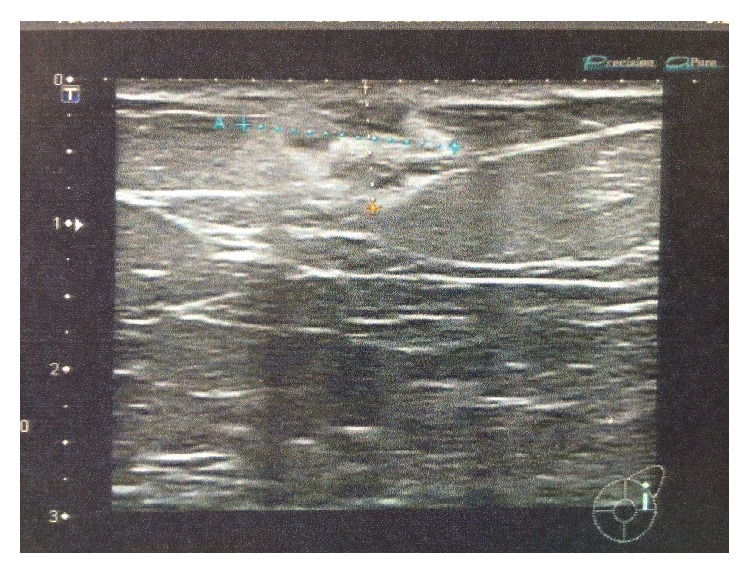
Ultrasound: heterogeneous oval mass with echogenic rim; BI-RADS ACR4.

A 67-year-old female patient was admitted to our breast unit for management of a recently palpated lump located on the upper outer quadrant of her left breast. Past medical history was significant for a total abdominal hysterectomy at the age of 51 due to multiple, large fibroids. She had no family history of breast cancer and previous screening mammograms were normal. Physical examination revealed a nontender, firm mass in the upper outer quadrant of the left breast with moderate skin dimpling. Examination of the contralateral breast and both axillas was unremarkable. Bilateral diagnostic mammogram (Figures 1 and 2) demonstrated a roughly round-shaped mass with ill-defined margins measuring 1 × 0.8 × 0.7 centimeters, situated on the upper outer quadrant of the left breast, without any other abnormality of the contralateral breast or both axillary regions. The lesion was reported as BIRADS IV; dedicated breast ultrasound confirmed a heterogeneous oval mass with echogenic rim, reported as BI-RADS ACR4 (Figure 3).

The patient underwent an excision biopsy under general anesthesia; on frozen section the tumor was highly suspicious for lymphatic or myofibroblastic neoplasia. Surgical margins of the specimen were free of disease; in view of the frozen section evaluation, planned sentinel lymph node biopsy was not performed.

**Figure 4:**
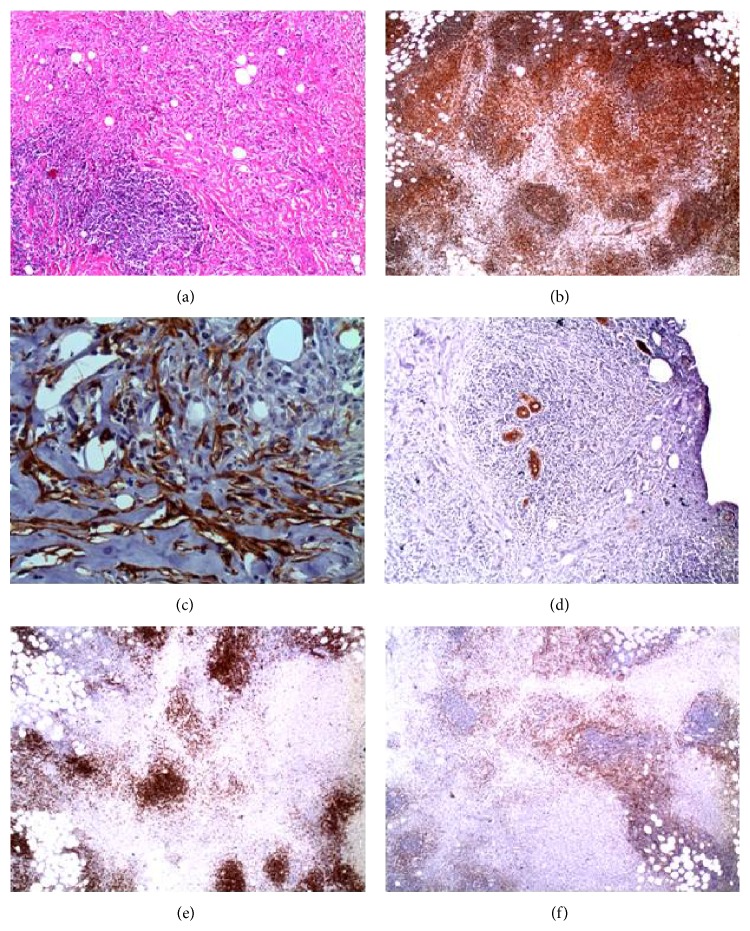
(a) Hematoxylin-eosin (H&E) staining of the lesion (magnification ×10): significant inflammatory invasion by lymphocytes and plasma cells; presence of stacks of fusiform myofibroblasts and few gigantic cells. (b) Vimentin staining of the lesion (magnification ×10): presence of myofibroblasts and lymphocytes. (c) Actin staining of the lesion (magnification ×40): presence of myofibroblasts which stain positive for actin. (d) Keratin 34BE12 staining (magnification ×10): myofibroblasts immunonegative to keratin 34BE12. (e) CD20 staining (magnification ×4): CD20 positive B-lymphocytes. (f) CD3 staining (magnification ×4): CD3 positive T-lymphocytes.

Pathology examination of paraffin embedded sections of the tumor exhibited tuberous aggregations of lymphocytes without any blastic centers, with concurrent invasion by numerous plasma cells, histiocytes and gigantic cells, probably myofibroblasts, and scarce polymorphonuclear and eosinophilic cells. At immunohistochemistry the lesion stained positive for vimentin, actin, plasmatocyte CD138, histiocyte CD68, Ki67 (5%), and lymphocytes CD20, CD3, and CD5, while it was negative for desmin, anaplastic lymphoma kinase (ALK), pankeratin, keratin 34BE12, CD15, and CD30. Pathology concluded for an anaplastic lymphoma kinase- (ALK-) negative inflammatory myofibroblastic tumor of the breast (Figures 4(a)–4(f)).

Full staging investigations were negative for systemic disease and the patient received no further treatment. Six months after surgery, the patient is well and free of disease.

## 3. Discussion

The World Health Organization (WHO) classifies inflammatory myofibroblastic tumors (IMTs) as distinct borderline lesions, while the physical history of these lesions can variably range from reactive to truly neoplastic [1]. Inflammatory myofibroblastic tumor of the breast is an extremely rare lesion. Generally, IMTs are roughly considered as a subset of inflammatory pseudotumors (IPTs); yet the interchangeable use of either term in the literature can generate some confusion; purportedly, these two entities (IMT and IPT) share common morphology although they demonstrate different clinicopathological features [9].

IMTs have been observed predominantly in young patients and have been encountered virtually in any anatomical location such as the lungs [2], mesentery [4], omentum [3], retroperitoneum [5], extremities [6], head [7], liver [10], spleen [11], thyroid [12], and urinary bladder [13]. Notably, reported cases on IMTs/IPTs of the breast are very scarce [1, 9, 14–34].

Microscopically, IMTs usually are characterized by the presence of spindle cell proliferation and inflammatory infiltrates by lymphocytes, plasma cells, histiocytes, or, less frequently, large vacuolated cells [15, 16]. The pathogenesis of IMTs is largely undetermined. While they were initially believed to generate upon an inflammatory or infectious stimulus, recent studies on these rare entities have revealed aberrations located in chromosomes 2 and 9 [35]. Furthermore, nearly half of IMT cases reportedly exhibit mutations involving the anaplastic lymphoma receptor tyrosine kinase gene (ALK) at 2p23. This feature has favored a neoplastic nature of these tumors, as clonal abnormalities of the ALK gene were first described in anaplastic large cell lymphoma (ALCL), which is a true neoplasia [1, 36, 37]. In ALCL patients though, ALK-positivity is reportedly accompanied by a less aggressive clinical course. Whether there is a more favorable outcome of patients with an ALK-positive IMT is yet to be defined [38, 39].

Since an IMT of the breast can mimic or even behave as true malignancy, wide excision with negative margins is highly recommended [1, 37]. As this is a very rare lesion to be encountered in the breast, data on malignant course, recurrence, and metastasis are scarce. Zhao et al. reported one patient who had local recurrence and metastasis to the left groin area 3, 7, and 10 months after initial surgery for an IMT of the breast [14]. Moreover, studies have reported on recurrence rates up to 25% for IMTs on other anatomical locations [37].

Interestingly, axillary management in cases of breast IMT is yet undefined. In none of the case reports so far did the authors encounter any axillary involvement in the setting of a primary IMT of the breast. Furthermore, the sarcomatous component of this lesion could raise a premise that lymphatic spread is unusual and hence routine SNLB is unnecessary. Still, larger series in the future could probably clarify this matter.

Chemotherapy, radiotherapy, and immunomodulation have not been reported to be consistently effective against IMTs, albeit sporadic cases of treatment with chemotherapy or anti-inflammatory agents have been reported [40, 41]. Wide excision with free margin remains the treatment of choice for patients with IMTs and is adequate in most circumscribed tumors. However, tumors with ill-defined morphology and/or incomplete resection have been associated with higher recurrence rates [42].

## 4. Conclusion

IMT of the breast represents a very rare entity with intermediate clinical behavior. Although reportedly infrequent, recurrence and metastatic potential exist and, as such, wide surgical resection with close clinical mammographic follow-up is recommended.

## References

[1] Bosse K., Ott C., Biegner T. (2014). 23-year-old female with an inflammatory myofibroblastic tumour of the breast: a case report and a review of the literature. *Geburtshilfe und Frauenheilkunde*.

[2] Sonomura T., Hasegawa S., Takeuchi H., Ishii S., Sato M. (2014). Inflammatory myofibroblastic tumor of the lung indistinguishable from adenocarcinoma on imaging studies. *Clinical Nuclear Medicine*.

[3] Kye B. H., Kim H. J., Kang S.-G., Yoo C., Cho H.-M. (2012). A case of inflammatory myofibroblastic tumor originated from the greater omentum in young adult. *Journal of the Korean Surgical Society*.

[4] Yagmur Y., Akbulut S., Gumus S. (2014). Mesenteric inflammatory pseudotumor: a case report and comprehensive literature review. *Journal of Gastrointestinal Cancer*.

[5] Chatzikokolis S., Troupis T. G., Michalinos A., Bafaloukas N., Filippidis T., Gennimatas V. (2012). Retroperitoneal inflammatory myofibroblastic tumor. *The American Surgeon*.

[6] Lin J., Liu H., Zhuang Y. (2014). Inflammatory myofibroblastic tumor of the thigh without bone involvement: a case report. *World Journal of Surgical Oncology*.

[7] Wang J., Sun Z., Zhuo S., Wang K. (2015). Sigmoid sinus occlusion infiltrated by inflammatory myofibroblastic tumor from mastoid. *Head & Neck*.

[8] Gilani S. M., Kowalski P. J. (2014). Inflammatory myofibroblastic tumour: a rare entity with wide differential diagnosis. *Pathologica*.

[9] Vecchio G. M., Amico P., Grasso G., Vasquez E., La Greca G., Magro G. (2011). Post-traumatic inflammatory pseudotumor of the breast with atypical morphological features: a potential diagnostic pitfall. Report of a case and a critical review of the literature. *Pathology Research and Practice*.

[10] Nagarajan S., Jayabose S., McBride W. (2013). Inflammatory myofibroblastic tumor of the liver in children. *Journal of Pediatric Gastroenterology and Nutrition*.

[11] Krl E. A., Orhan D., Haliloğlu M., Karnak I. (2012). Invasive inflammatory myofibroblastic tumor of the spleen treated with partial splenectomy in a child. *Journal of Pediatric Hematology/Oncology*.

[12] Trimeche M., Ziadi S., Mestiri S. (2009). Inflammatory myofibroblastic tumor of the thyroid in its sclerosing subtype: the first case report. *European Archives of Oto-Rhino-Laryngology*.

[13] Dobrosz Z., Ryś J., Paleń P., Właszczuk P., Ciepiela M. (2014). Inflammatory myofibroblastic tumor of the bladder—an unexpected case coexisting with an ovarian teratoma. *Diagnostic Pathology*.

[14] Zhao H.-D., Wu T., Wang J.-Q. (2012). Primary inflammatory myofibroblastic tumor of the breast with rapid recurrence and metastasis: a case report. *Oncology Letters*.

[15] Zhou Y., Zhu J., Zhang Y., Jiang J., Jia M. (2013). An inflammatory myofibroblastic tumour of the breast with ALK overexpression. *BMJ Case Reports*.

[16] Gobbi H., Atkinson J. B., Kardos T. F., Simpson J. F., Page D. L. (1999). Inflammatory myofibroblastic tumour of the breast: report of a case with giant vacuolated cells. *Breast*.

[17] Akbulut M., Gunhan-Bilgen I., Zekioglu O., Duygulu G., Oktay A., Ozdemir N. (2007). Fine needle aspiration cytology of inflammatory myofibroblastic tumour (inflammatory pseudotumour) of the breast: a case report and review of the literature. *Cytopathology*.

[18] Sastre-Garau X., Couturier J., Derré J., Aurias A., Klijanienko J., Lagacé R. (2002). Inflammatory myofibroblastic tumour (inflammatory pseudotumour) of the breast. Clinicopathological and genetic analysis of a case with evidence for clonality. *The Journal of Pathology*.

[19] Zardawi I. M., Clark D., Williamsz G. (2003). Inflammatory myofibroblastic tumor of the breast. A case report. *Acta Cytologica*.

[20] Khanafshar E., Phillipson J., Schammel D. P., Minobe L., Cymerman J., Weidner N. (2005). Inflammatory myofibroblastic tumor of the breast. *Annals of Diagnostic Pathology*.

[21] Ilvan S., Celik V., Paksoy M., Cetinaslan I., Calay Z. (2005). Inflammatory myofibroblastic tumor (inflammatory pseudotumor) of the breast. *APMIS*.

[22] Li J., Yun W., Qin J. (2013). Inflammatory myofibroblastic tumor of the breast coexisting with breast cancer: a case report. *Breast Care*.

[23] Nomelini R. S., Jammal M. P., Fernandes P. C., de Carvalho Mardegan M., Saldanha J. C., Murta E. F. C. (2011). Myofibroblastic inflammatory breast tumor and fibrosarcoma: a case report. *European Journal of Gynaecological Oncology*.

[24] Park S. B., Kim H. H., Shin H. J., Gong G. (2010). Inflammatory pseudotumor (myoblastic tumor) of the breast: a case report and review of the literature. *Journal of Clinical Ultrasound*.

[25] Kim S. J., Moon W. K., Kim J. H., Cho N., Chang C. M. (2009). Inflammatory pseudotumor of the breast: a case report with imaging findings. *Korean Journal of Radiology*.

[26] Hill P. A. (2010). Inflammarory pseudotumor of the breast: a mimic of breast carcinoma. *Breast Journal*.

[27] Pettinato G., Manivel J. C., Insabato L., Chiara De A., Petrella G. (1988). Plasma cell granuloma (inflammatory pseudotumor) of the breast. *The American Journal of Clinical Pathology*.

[28] Yip C. H., Wong K. T., Samuel D. (1997). Bilateral plasma cell granuloma (inflammatory pseudotumour) of the breast. *The Australian and New Zealand Journal of Surgery*.

[29] Chetty R., Govender D. (1997). Inflammatory pseudotumor of the breast. *Pathology*.

[30] Haj M., Weiss M., Loberant N., Cohen I. (2003). Inflammatory pseudotumor of the breast: case report and literature review. *The Breast Journal*.

[31] Sciallis A. P., Chen B., Folpe A. L. (2012). Cellular spindled histiocytic pseudotumor complicating mammary fat necrosis: a potential diagnostic pitfall. *The American Journal of Surgical Pathology*.

[32] Sari A., Yigit S., Peker Y., Morgul Y., Coskun G., Cin N. (2011). Inflammatory pseudotumor of the breast. *The Breast Journal*.

[33] Zen Y., Kasahara Y., Horita K. (2005). Inflammatory pseudotumor of the breast in a patient with a high serum IgG4 level: histologic similarity to sclerosing pancreatitis. *The American Journal of Surgical Pathology*.

[34] Nori J., Masi A., Vivian A., di Lollo S., Boeri C. (2000). Inflammatory pseudotumor of the breast. A case report. *La Radiologia Medica*.

[35] Griffin C. A., Hawkins A. L., Dvorak C., Henkle C., Ellingham T., Perlman E. J. (1999). Recurrent involvement of 2p23 in inflammatory myofibroblastic tumors. *Cancer Research*.

[36] Downing J. R., Shurtleff S. A., Zielenska M. (1995). Molecular detection of the (2;5) translocation of non-Hodgkin's lymphoma by reverse transcriptase-polymerase chain reaction. *Blood*.

[37] Gleason B. C., Hornick J. L. (2008). Inflammatory myofibroblastic tumours: where are we now?. *Journal of Clinical Pathology*.

[38] Falini B., Pileri S., Zinzani P. L. (1999). ALK^+^ lymphoma: clinico-pathological findings and outcome. *Blood*.

[39] Gascoyne R. D., Aoun P., Wu D. (1999). Prognostic significance of anaplastic lymphoma kinase (ALK) protein expression in adults with anaplastic large cell lymphoma. *Blood*.

[40] Dishop M. K., Warner B. W., Dehner L. P. (2003). Successful treatment of inflammatory myofibroblastic tumor with malignant transformation by surgical resection and chemotherapy. *Journal of Pediatric Hematology/Oncology*.

[41] Bonnet J. P., Basset T., Dijoux D. (1996). Abdominal inflammatory myofibroblastic tumors in children: report of an appendiceal case and review of the literature. *Journal of Pediatric Surgery*.

[42] Lu C.-H., Huang H.-Y., Chen H.-K., Chuang J.-H., Ng S.-H., Ko S.-F. (2010). Huge pelvi-abdominal malignant inflammatory myofibroblastic tumor with rapid recurrence in a 14-year-old boy. *World Journal of Gastroenterology*.

